# CanCPLD: Convective Parameters and Lightning Data to Support Future Thunderstorm Projections in North America

**DOI:** 10.1038/s41597-025-05924-7

**Published:** 2025-10-10

**Authors:** Alex J. Cannon, Kathleen S. Ramsey, Alessio C. Spassiani

**Affiliations:** 1 Climate Research Division, Environment and Climate Change Canada, Victoria, BC Canada; 2 Department of Physics & Astronomy, University of British Columbia, Vancouver, BC Canada; 3 Climate Research Division, Environment and Climate Change Canada, Toronto, ON Canada

**Keywords:** Natural hazards, Projection and prediction

## Abstract

Thunderstorms cause natural hazards, including hail, floods, strong winds, and lightning. Simulating thunderstorms in climate models is challenging due to their small scale, the complexity of their physical drivers, and the need to parameterize subgrid processes. Thunderstorm activity can be inferred by identifying relevant historical environmental parameters, e.g. convective available potential energy, humidity, and wind shear, and building statistical models that use these parameters as proxies for thunderstorm occurrence. Climate model projections of the parameters can be used with the statistical models to assess future thunderstorm activity. In this context, a multi-decade dataset with lightning flash totals and 201 convective parameters has been compiled for North America, focusing on areas north of 40°N. Parameters from the European Centre for Medium-Range Weather Forecasts reanalysis version 5 are available at 3-hour intervals on a 0.25° grid. The same variables are calculated for HighResMIP climate model simulations at 6-hour intervals for the historical period coinciding with global warming of 1°C above preindustrial and future periods at 2°C, 3°C, and 4°C warming.

## Background & Summary

Thunderstorms can cause severe weather and natural hazards, such as hail, heavy rain, straight line winds, and tornadoes^1^, as well as lightning strikes^2^ and wildfire ignitions^3,4^. These hazards pose substantial risks to life, property, and ecosystems, and therefore there is increasing interest in understanding how the frequency, intensity, and spatial distribution of thunderstorms will respond to anthropogenic climate change^5,6^. Accurate simulation of thunderstorms by climate models is particularly challenging because of their small scale – operating in the meso-*γ* storm scale regime (2-20 km) – and the complexity of physical processes involved in their initiation, maturation, and dissipation. Climate models typically operate at a spatial resolution of hundreds to many tens of kilometers, which is insufficient to explicitly simulate deep moist convection in thunderstorms. Although convection-permitting climate models (~4 km grid spacing or finer) allow explicit simulation of some convective processes without the need for parameterization^7^, they are computationally demanding and hence are often limited in their temporal and spatial coverage^6^. Instead, changes in thunderstorm activity over larger areas and longer simulations can be inferred by identifying relevant environmental conditions on resolved scales, such as convective available potential energy (CAPE), convective inhibition (CIN), lifting condensation level (LCL), humidity, vertical wind shear, storm motion, and other indicators of the convective storm environment, from modern atmospheric reanalyses, such as the European Centre for Medium-Range Weather Forecasts (ECMWF) reanalysis version 5 (ERA5)^8^. Statistical or machine learning techniques can be used to relate these proxy variables to thunderstorm activity (e.g., from lightning^9^ or severe weather reports^10,11^). Once derived, these empirical relationships can, assuming stationarity, be used in conjunction with the projections of proxy conditions in the climate model^12^ to infer potential changes in thunderstorm activity^13^.

Most studies linking the convective environment with thunderstorm activity in North America^1,6,14^ have focused on the continental United States (US). This is mainly due to the relatively high frequency of severe thunderstorms, centred in the southeastern and south-central US^15^, and the availability of long records of observer reports of thunderstorm occurrence and data from automated lightning detection sensors^16,17^. Fewer studies have focused on the high latitudes of North America^18^, here defined as the area north of 40°N^19^. Although less frequent in this region^20,21^, thunderstorm hazards are still responsible for loss of life and property damage^22,23^. Furthermore, northern land areas have experienced and are projected to continue to experience more rapid warming than the global average due to Arctic amplification^24,25^, raising the possibility of a northerly expansion of convective environments that are favourable for the development of thunderstorms with continued climate change^12^.

The modelling chain – moving from convective parameters to predictions of thunderstorm activity – requires historical thunderstorm observations^26^, three-dimensional (3D) atmospheric state variables necessary to compute convective storm parameters associated with observed storms, and finally climate model simulations of these same variables at comparable spatiotemporal scales. In most of northern North America, the surface observational network is less dense than in the contiguous US, which means that observer reports of tornadoes, waterspouts, funnel clouds, thunderstorms, and lightning may only be able to support modelling studies in populated regions of the country. However, lightning strike data from automated lightning detection sensors in Environment and Climate Change Canada’s (ECCC’s) Canadian Lightning Detection Network (CLDN), which covers the high latitudes of North America, have now been available for more than 25 years^21^, which is sufficient to train statistical and machine learning models.

To obtain historical environmental parameters, atmospheric data should be available at a subdaily time step, a horizontal grid spacing of tens of kilometers or less, and with sufficient vertical resolution to accurately calculate parameters such as CAPE, CIN, and vertical wind shear. Modern reanalyses have the potential to supply these data, and their suitability for use in thunderstorm modelling has been evaluated in recent studies^14,27,28^.The findings indicate that ERA5, in part due to its higher resolution (archived at an hourly time step on a 0.25° grid), is probably the most reliable reanalysis currently available to study convective storm environments. However, because convective indices are derived from the full 3D thermodynamic and kinematic state of the atmosphere, their calculation at this spatiotemporal resolution requires a time- and resource-intensive network transfer, preprocessing, computing, and validation chain of operations. This poses a practical barrier for many researchers working on local workstations, shared servers, or metered cloud platforms where network bandwidth, storage and computing quotas, and egress charges are limiting. By distributing a precomputed archive of convective parameters, the goal is to remove this data transfer and computational burden and lower the entry threshold for thunderstorm research.

As noted earlier, the grid spacing of most global climate models has, historically, been much coarser than that of ERA5 and hence the utility of global models for regional studies of convective storms has been questioned. As an alternative, the North American Coordinated Regional Downscaling Experiment (NA-CORDEX) includes regional model output with comparable spatial resolution^29^. However, simulations are not yet widely available for the most recent phase of the Coupled Model Intercomparison Project (CMIP6) and 3D state variables are only available by request from each participating modelling centre. Within CMIP6, an endorsed model intercomparison project, HighResMIP^30^, organized simulations from high-resolution global climate models with horizontal and vertical resolutions similar to ERA5, either in atmosphere-only (AGCM) or coupled atmosphere-ocean (AOGCM) configurations. Unlike most of the CMIP6 archive, these outputs are well suited to investigate future thunderstorm environments. However, data input/output, processing, and validation issues noted above for ERA5 are amplified when dealing with centennial-scale climate model simulations, especially when using an ensemble of models to account for structural model uncertainty.

Taking these points into account, CanCPLD, a comprehensive multidecade dataset (1998-2024 in ERA5 and four 20-year periods in HighResMIP models), has been compiled to support studies on the future evolution of thunderstorms in North America. Data included in CanCPLD are summarized in Table 1. The dataset includes 3-hourly cloud-to-ground (CG) lightning flash totals, and for completeness, intra-cloud/cloud-to-cloud CC flashes, from the CLDN (regions north of 40°N on a 0.1° grid), as well as 201 convective storm parameters derived from ERA5 outputs (3-hour intervals for all of North America on a 0.25° grid). Additionally, the same parameters are calculated from HighResMIP climate model simulations at 6-hour intervals for 20-year periods corresponding to 1°C (recent past) and 2°C, 3°C, and 4°C levels of global warming above the preindustrial mean.

**Table 1:** Gridded data used to calculate lightning data (LD) and convective parameters (CP) in the CanCPLD dataset.

Identifier	Member	Type	Period(s)	Grid	Levels	Time step
CLDN^21^ (LD)	—	Observed	1998-2024	0. 1° × 0. 1°	—	3-hourly
ERA5^8^ (CP)	—	Reanalysis	1998-2024	0.25° × 0.25°	18	3-hourly
CMCC-CM2-VHR4^60^ (CP)	r1i1p1f1	AOGCM	+1°C to 2°C	~0.31° × 0.23°	8	6-hourly
EC-Earth3P-HR_r1^61^ (CP)	r1i1p2f1	AOGCM	+1°C to 2°C	~0.35° × 0.35°	18	6-hourly
EC-Earth3P-HR_r2^61^ (CP)	r2i1p2f1	AOGCM	+1°C to 2°C	~0.35° × 0.35°	18	6-hourly
EC-Earth3P-HR_r3^61^ (CP)	r3i1p1f1	AGCM	+1°C to 2°C	~0.35° × 0.35°	8	6-hourly
MRI-AGCM3-2-H^62,63^ (CP)	r1i1p1f1	AGCM	+1°C to 4°C	~0.56° × 0.56°	8	6-hourly
MRI-AGCM3-2-S^63,64^ (CP)	r1i1p1f1	AGCM	+1°C to 4°C	~0.19° × 0.19°	8	6-hourly

LD cover the high latitude regions of North America (north of 40°N). The CP data cover all of North America.

18 levels: surface and 1000-, 975-, … , 800-, 750-, 700-, 600-, … , 100-hPa.

8 levels: surface and 925-, 850-, 700-, 600-, 500-, 250-, 50-hPa.

## Methods

### Lightning data

Gridded lightning data (Table 1) are derived from CLDN observations. The CLDN has been operational since 1998, providing a comprehensive national system to detect the time, location, amplitude, and polarity of lightning in Canada and adjacent areas of the US. Initially comprising 81 sensors, the network has undergone continuous upgrades that have improved detection efficiency (from 90% to 95% for CG strokes) and location accuracy (now within 250 m). The CLDN is an integral part of the North American Lightning Detection Network^31^, leveraging Vaisala sensors in the northern US to improve detection in southern Canada. Beginning in early 1999, the CLDN detects both CC and CG flashes and strokes, with a flash consisting of one or more strokes. Detection efficiency for CG strokes has remained high over time, but the efficiency for CC lightning has improved from 10% to 50%, introducing temporal inhomogeneities in the CC data. Furthermore, improvements to the Vaisala processing system in early November 2017 have led to better classification of low-current positive CC strokes; up until this date, strokes were often misclassified as CG lightning^32^.

**Fig. 1:**
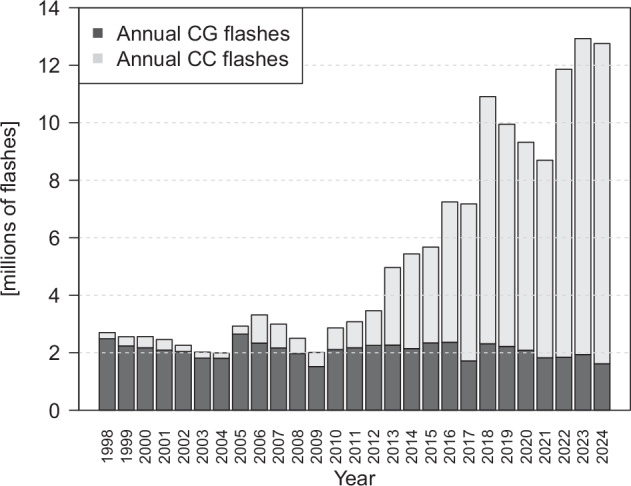
Total lightning flashes over Canada categorized as CG and CC per year.

CLDN data in CanCPLD are restricted to lightning flash counts, which are derived from processed lightning flash data received by ECCC after the end of each month from Vaisala. Following established practice^21,21,33^, these data have undergone additional processing to reclassify low-current CG+ flashes before early November 2017 (<15 kA) as CC flashes, and high current CC+ flashes (>20 kA) as CG flashes. For reference, Fig. 1 compares annual total CG and CC flashes over Canada; the increasing trend in CC flashes, due to improvements in detection efficiency, is clear. Information on the suitability of the data for trend analysis – as well as information on polarity and magnitude – has been summarized in past studies^21,33– 35^. However, given the inhomogeneity of the CC flash data and the relatively stable detection efficiency of CG flashes (>90%), it is recommended that CG flashes be used for climatological thunderstorm analyses.

Gridded CG counts in CanCPLD include flashes from convective cores and associated stratiform/anvil regions. In organized convection, anvils often extend on the order of 100 km downshear and can produce CG lightning tens to >100 km from the convective core^36–38^. Consequently, CG lightning during an event should be interpreted as the total lightning footprint of the storm system, not solely the convective core. However, case studies of midlatitude mesoscale convective systems show that stratiform/anvil regions contribute a minority of storm lightning^39^; for example, in a leading-line/trailing-stratiform system, the convective line produced about 12 times more CG lightning than the stratiform region^40^. Most CG lightning remains confined to convective cores^41^.

Although separation into CG and CC flashes formally began in February 1999, CanCPLD provides CG and CC flash counts starting in 1998. For months before February 1999, all flashes are first assumed to be CG, with CC flashes in the early part of the record appearing solely because of the reclassification of low-current positive flashes noted above. The total numbers of lightning flashes that occur within regular 0.1° grid cells (north of 40°N) are counted for 3-hour periods beginning at 00:00 UTC on January 1, 1998 until the end of 2024. Grid cell areas (m^2^) are computed from the cell bounds on a spherical Earth, which inherently accounts for meridian convergence, and are provided to allow lightning flash totals to be normalized into lightning flash densities. For context, at this resolution a 0.1° grid cell has an area of around 95 km^2^ at 40°N and 62 km^2^ at 60°N.

### ERA5 reanalysis

Historical convective parameters are calculated using 3D atmospheric state variables from the ERA5 reanalysis dataset^8^ (Table 1) for the same period of record as the lightning flash data (1998-2024). ERA5, produced by the European Centre for Medium-Range Weather Forecasts (ECMWF), is a fifth-generation global atmospheric reanalysis that provides data on a 0.25° (~31 km) grid and with a temporal resolution of 1 hour. ERA5 data have been retrieved from the Copernicus Climate Data Store and processed to extract the required variables over North America (15°N-90°N, 180°W-40°W). Data are obtained at a 3-hour time step to balance temporal detail against computational cost and storage volume.

Although ERA5 provides air temperature, geopotential height, specific humidity, and horizontal wind components on 37 standard pressure levels plus the surface, a subset of 17 pressure levels plus the surface is used here for computational efficiency and compatibility with global climate model projections. This subset spans the lower to upper troposphere, including the same levels as the entire ERA5 dataset from the surface up to 800 hPa, with a reduced vertical resolution above 800 hPa. The selected levels are 1000, 975, 950, 925, 900, 875, 850, 825, 800, 750, 700, 600, 500, 400, 300, 200, and 100 hPa, as well as the surface. Reducing the number of vertical levels minimizes data storage requirements and computational costs while retaining sufficient resolution for reasonably accurate calculations of convective parameters such as CAPE, CIN, and vertical wind shear. Furthermore, the reduced set of pressure levels aligns with outputs from HighResMIP global climate models, which typically provide data for fewer pressure levels than ERA5. This ensures consistency when comparing historical reanalysis data with projections. The impact of the reduction in vertical levels is assessed in the subsequent technical validation.

### HighResMIP simulations

To complement the ERA5 reanalysis data, historical and future simulations from a subset of HighResMIP global climate models^30^ are used to calculate convective parameters (Table 1). Data have been downloaded from the Earth System Grid Federation climate data archive^42^. Two experimental configurations are included in CanCPLD: atmosphere-only (AGCM) simulations, which use prescribed sea surface temperatures and sea ice concentrations derived from RCP8.5 output; and coupled atmosphere-ocean (AOGCM) simulations. All simulations extend at least to 2050, with some modelling centres providing data through 2100, enabling an exploration of storm environments under both near-term and long-term future climate conditions. External forcings, including greenhouse gas concentrations and aerosols, follow the SSP5-8.5 scenario, which is roughly consistent with the RCP8.5 scenario used for the prescribed sea surface temperatures and sea ice concentrations in the atmosphere-only simulations.

The selection of HighResMIP simulations is guided by several criteria to ensure fairly uniform spatial and temporal resolution, thus establishing a consistent framework for assessing the evolution of convective storm environments under historical and future climate scenarios, as well as observational estimates from ERA5. Only models with a horizontal grid spacing of approximately 0.5° or finer and a complete set of subdaily 3D fields are included, roughly aligning with the spatial and temporal resolution of the ERA5 output. The temporal frequency of the HighResMIP data is 6-hourly, which is the highest time resolution available for most models, in contrast to the 3-hourly intervals used for ERA5. Atmospheric state variables are conservatively remapped to the same North American spatial domain and grid as ERA5. The number of vertical levels archived for the HighResMIP simulations depends on the model; some include fewer pressure levels than ERA5 (Table 1).

Because climate models have varying sensitivities to external forcings^43^, their outputs are organized based on global warming levels (GWLs) relative to preindustrial conditions. However, HighResMIP simulations start in 1950 rather than in the preindustrial period. Therefore, it is assumed that the +1°C GWL is reached in all models during a time frame coinciding with observational estimates (2001-2020)^44^. Based on this reference point, model outputs for 20-year periods corresponding to +2°C, +3°C, and +4°C of warming above preindustrial levels are identified and extracted. All models reach the +2°C GWL (an additional 1°C of warming beyond the +1^∘^C baseline) by 2050. For models that extend to 2100, results for the +3°C and +4°C GWLs are also available.

### Convective parameters

Convective parameters in CanCPLD are calculated by the sounding_compute routine from the thundeR R package (v1.1.3)^45,46^ applied to 3D atmospheric state variables from ERA5 and the HighResMIP climate model simulations. Default arguments are adopted for vertical interpolation accuracy and heights of the layer used to compute parcel starting parameters. The routine provides a comprehensive set of 201 convective parameters that collectively describe the thermodynamic and kinematic properties of the atmosphere, capturing conditions conducive to the development of deep moist convection. Key parameters include different formulations (most unstable, MU; surface-based, SB; and mixed layer, ML) of CAPE, CIN, LCL, lifted index, and level of free convection, as well as measures of wind shear, lapse rate, atmospheric moisture, storm-relative helicity, and storm motion. The full list of parameters and their units is provided in Supplementary Table 1.

## Data Records

CanCPLD^47^ is available from the ECCC Data Catalogue with identifier 10.18164/9a68a501-9a87-441a-8073-980ae68438efand also from the Government of Canada Open Data Portal (https://open.canada.ca/data/en/dataset/9a68a501-9a87-441a-8073-980ae68438ef). The dataset is released under the Open Government Licence - Canada, which grants users a worldwide, royalty-free, perpetual, non-exclusive licence to use the data, including for commercial purposes, subject to terms listed at https://open.canada.ca/en/open-government-licence-canada.

CanCPLD consists of three components: gridded CLDN lightning flash data, gridded ERA5 convective parameters, and gridded HighResMIP convective parameters. Data are in self-describing, binary netCDF files organized for download in sub-directories as follows:

- CLDN/:
  - cc/: cc_flashes_3hr_0.1-deg_1998.nc, … , cc_flashes_3hr_0.1-deg_2024.nc
    Each netCDF file contains 3-hourly CC lightning flash counts starting at times 00:00, 03:00, … , and 21:00 UTC with variable name and dimensions of cc_flashes(time, lat, lon) for 0.1° × 0.1° grid cells in the given year. The region outside the area of coverage of the CLDN is masked.
  - cg/: cg_flashes_3hr_0.1-deg_1998.nc, … , cg_flashes_3hr_0.1-deg_2024.nc
    As for cc/, but containing CG lightning flash counts with variable name and dimensions of cg_flashes(time, lat, lon).
  - fixed/: cell_area_0.1-deg.nc
    A netCDF file with the area (m^2^) of each 0.1° grid cell with variable name and dimensions of area(lat, lon).
- ERA5/:
  - 1998/: 19980101.nc, … , 19981231.nc
    … 
  - 2024/: 20240101.nc, … , 20241231.nc
    Each netCDF file contains values of ERA5 convective parameters for the given date at times 00:00, 03:00, … , and 21:00 UTC with variable name and dimensions of *I*(time, latitude, longitude), where *I* corresponds to each of the 201 convective parameters listed in Supplementary Table 1. Data are on a 0.25° grid over the spatial domain from 180°W to 40°W and 15°N to 90°N.
  - GWL1.0/ :
    ∗ 2001/: 20010101.nc, … , 20011231.nc
    … 
    ∗ 2020/: 20200101.nc, … , 20201231.nc
    As above for 1998-2024, but limited to years corresponding to the +1°C GWL above preindustrial (GWL1.0; 2001-2020).
- EC-Earth3P-HR_r1/, …, EC-Earth3P-HR_r3/, MRI-AGCM3-2-H/, MRI-AGCM3-2-S/
  - GWL1.0/
    ∗ YR01/: YR010101, … , YR011231.nc
    … 
    ∗ YR20/: YR200101, … , YR201231.nc
  - GWL2.0/
    ∗ YR01/: YR010101, … , YR011231.nc
    … 
    ∗ YR20/: YR200101, … , YR201231.nc
  - GWL3.0/ and GWL4.0/ if available.

Each netCDF file contains values of convective parameters for the specified HighResMIP simulation and date at times 00:00, 06:00, … , and 18:00 UTC with variable name and dimensions of *I*(time, latitude, longitude), where *I* corresponds to each of the 201 convective parameters listed in Supplementary Table 1. Data are on a 0.25° grid over a spatial domain from 180°W to 40°W and 15°N to 90°N. The netCDF files are organized in sub-directories corresponding to the HighResMIP simulation and years (YR01, YR02, … , YR20) associated with each GWL (+1°C or GWL1.0 and  +2°C or GWL2.0, as well as +3°C or GWL3.0 and +4°C or GWL4.0 if reached).

## Technical Validation

### Lightning data

**Fig. 2:**
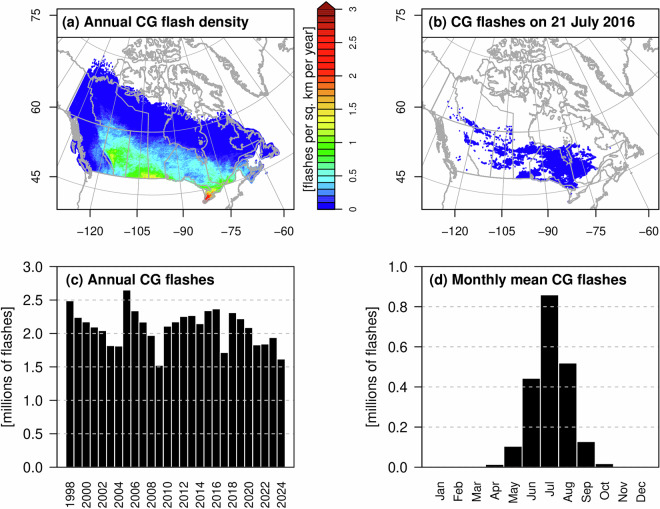
(**a**) Long-term annual mean CG flash density over Canada, (**b**) CG flashes on 21 July 2016, (**c**) total CG flashes per year, and (**d**) long-term monthly mean CG flashes. Each panel is an independent replication of results provided in the study by KB2020^21^.

The technical validity of the CanCPLD lightning flash data is checked by replicating previously published summaries of long-term climatological CG flash densities, extreme daily lightning activity, annual flash counts, and monthly flash counts for Canada^21^. Results are shown in Fig. 2. The overall spatial distribution of CG flash density is consistent with previous maps (cf. Fig. 4)^21^, with higher densities in southern Ontario, the Prairie provinces, and along the eastern slopes of the Rocky Mountains, and decreasing flash densities moving towards the northern latitudes. CG flashes on the most active lightning day in Canada (21 July 2016) (cf. Fig. 11)^21^ are also the same, with concentrated activity in western Quebec and Ontario that extends westward over the Prairies into northern British Columbia and eastern Yukon. Taking into account differences in spatial aggregation (0.1° grid in CanCPLD) and geographic masking, the annual CG flash totals and the monthly distribution of flashes closely resemble those previously reported (cf. Fig. 2)^21^.

### Convective parameters

#### ERA5

Previous studies have evaluated the representation of convective parameters in ERA5, including comparisons with parameters calculated from other reanalyses, rawinsondes and high-resolution Weather Research and Forecasting (WRF) model simulations^27,28,48,49^. The analysis here focuses on evaluating the impact of reducing the number of vertical levels used as inputs to sounding_compute on fidelity of the resulting convective parameters. Compared to CanCPLD, the 37 pressure levels available in ERA5 provide information on additional pressure levels between 800 hPa and 1 hPa. For most convective parameters, especially ones calculated using atmospheric variables at specific pressure levels, these additional levels will provide little to no benefit. However, those that involve vertical integrals through layers of the atmosphere, for example CAPE or CIN, may be degraded.

**Fig. 3:**
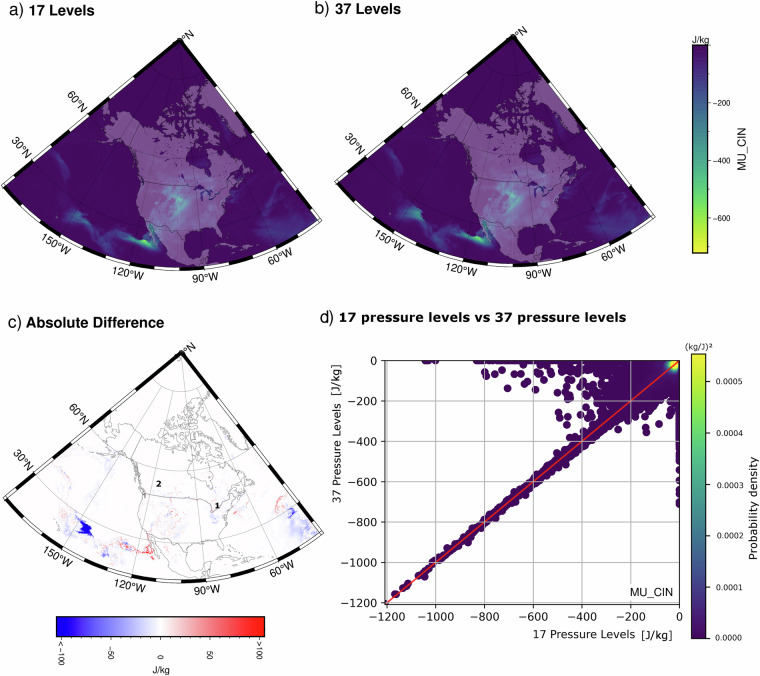
Mean values of MU_CIN for 21 July 2016 when calculated using (**a**) the subset of 17 CanCPLD pressure levels and (**b**) all 37 ERA5 pressure levels. (**c**) Daily mean absolute difference between the two calculations at each grid cell (panel b minus panel a). Locations marked 1 and 2 show the grid cells corresponding to the time series shown in Figs. 4 and 5, respectively. (**d**) Scatter plots comparing MU_CIN based on 17 and 37 pressure levels; colours indicate the density of overlapping values.

To evaluate, convective parameters are recalculated using all 37 levels at 3-hour time intervals on 21 July 2016 (see Fig. 2b)^21^. Table 2 summarizes spatial correlations, grid cell differences, and root mean squared errors between the two sets of calculations for a subset of convective parameters. For the North American domain as a whole, convective parameters calculated using 17 pressure levels are unbiased and highly correlated with those based on 37 levels. All spatial correlations are at least 0.95. For reference, daily mean values of MU_CIN, the parameter with the lowest spatial correlation, are compared in Fig. 3a–c. Differences are visually indistinguishable over land. Figure 3d plots grid cell values of MU_CIN calculated using 17 pressure levels against values calculated using all 37 pressure levels. Almost all values fall along the 1-to-1 line; only a small number of grid cells show large deviations. Spatially, the largest differences appear to cluster in the subtropics over the oceans (Fig. 3c).

**Table 2:** Comparison between convective parameters on 21 July 2016 calculated using 17 CanCPLD pressure levels (*I*_17_) and all 37 ERA5 pressure levels (*I*_37_); r is correlation, RMSE is root mean squared error, and differences are taken as *I*_37_ − *I*_17_.

Convective parameter (units)	Pearson r	RMSE	Difference (min.)	Difference (mean)	Difference (max.)
BS_02km (m s^−1^)	0.999	0.1	−4.5	0.0	4.2
BS_06km (m s^−1^)	0.998	0.3	−5.5	0.0	5.1
DCAPE (J kg^−1^)	0.999	16	−157	−5	140
MU_CAPE_500 (J kg^−1^)	0.999	21	−885	−16	912
MU_CAPE (J kg^−1^)	0.999	26	−3086	−15	2911
MU_CIN (J kg^−1^)	0.950	3	−711	−1	1043
MU_LFC_TEMP (J kg^−1^)	0.977	<1	−83	0	65
RH_01km (%)	0.999	<1	−16	0	16
RH_14km (%)	0.998	1	−10	0	9
RH_36km (%)	0.996	2	−16	0	13
RH_HGL (%)	0.996	2	−17	−1	15
SRH_3km_RM (m^2^ s^−2^)	0.998	2	−140	0	104

**Fig. 4:**
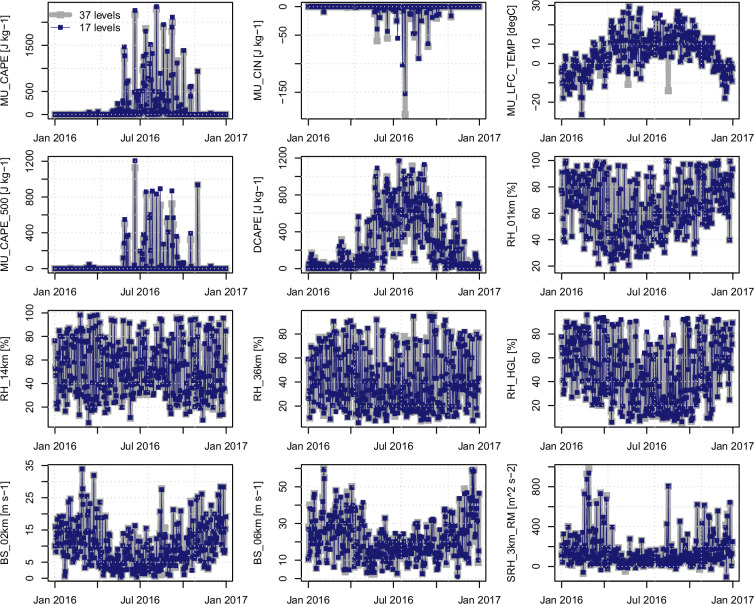
Time series of selected convective indices (21:00 UTC each day) for the location in southern Ontario (marked as 1 in Fig. 3c) for the year 2016.

**Fig. 5:**
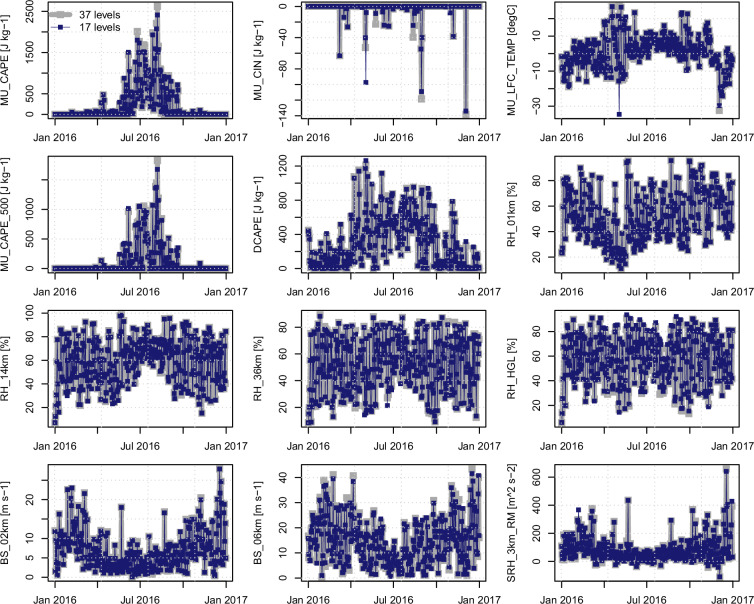
As in Fig. 4, but for the location east of the Rockies (marked as 2 in in Fig. 3c).

To extend the single-day evaluation for the North American domain to longer periods, daily time series (mid or late afternoon; 21:00 UTC) between convective parameters calculated using 17 and 37 pressure levels are compared at grid cells in southern Ontario and east of the Rockies (see Fig. 3c for grid cell locations). Performance statistics are shown in Table 3 for summer seasons over the period of record; time series for the entire 2016 year are shown in Figs. 4 and 5. Across both sites, parameters computed with the reduced set of 17 pressure levels closely track those computed with all 37 levels, with episodic deviations concentrated in MU_CIN and MU_LFC_TEMP. These two fields are especially sensitive to the choice of the most unstable parcel and to small vertical sampling differences that affect LCL/LFC placement; other thermodynamic, moisture, and shear variables show very similar behaviour between the full and reduced sets.

**Table 3:** Comparison between time series (21:00 UTC) of convective parameters calculated using 17 CanCPLD pressure levels (*I*_17_) and all 37 ERA5 pressure levels (*I*_37_) at grid cells in southern Ontario and east of the Rockies (locations [1] and [2], respectively, in the table headers and Fig. 3c).

Convective parameter (units)	Pearson r [1]	Pearson r [2]	RMSE [1]	RMSE [2]	Diff. (mean) [1]	Diff. (mean) [2]
BS_02km (m s^−1^)	0.995	0.993	0.4	0.3	0.0	0.0
BS_06km (m s^−1^)	0.993	0.993	0.8	0.8	0.0	0.0
DCAPE (J kg^−1^)	0.995	0.997	28	22	−8	−8
MU_CAPE_500 (J kg^−1^)	0.998	0.998	31	29	−12	−8
MU_CAPE (J kg^−1^)	0.998	0.998	44	50	−18	−2
MU_CIN (J kg^−1^)	0.825	0.923	13	7	−1	0
MU_LFC_TEMP (J kg^−1^)	0.900	0.902	3	2	0	0
RH_01km (%)	1.000	1.000	0	0	0	0
RH_14km (%)	0.996	0.993	2	2	0	0
RH_36km (%)	0.997	0.995	2	2	0	0
RH_HGL (%)	0.996	0.995	<1	<1	0	0
SRH_3km_RM (m^2^ s^−2^)	0.998	0.993	4	6	0	1

Statistics are calculated for summer seasons over the period of record; r is correlation, RMSE is root mean squared error, and differences are taken as *I*_37_ − *I*_17_.

#### HighResMIP

Convective parameters calculated from historical simulations of CMIP6 climate models exhibit some notable differences compared to those from ERA5 over North America^50^. However, all of the 12 global models considered in the evaluation study have horizontal grid spacings coarser than ERA5, with only one providing output on a grid finer than 1° × 1°. The HighResMIP simulations in CanCPLD are much more highly resolved, with most approaching or exceeding the horizontal grid spacing of ERA5 (Table 1).

**Fig. 6:**
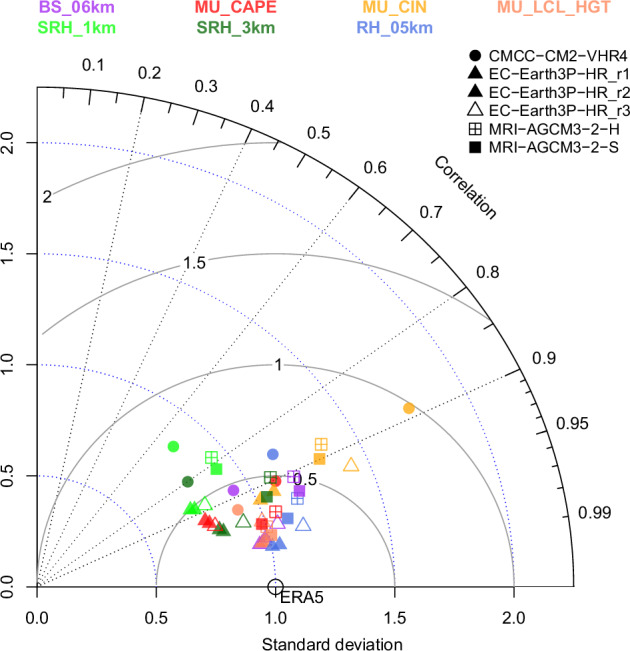
Taylor diagram summarizing HighResMIP model performance for historical simulations (+1°cC GWL; 2001-2020) of convective parameters over North American land areas. The plot shows spatial correlations (right curved axis; black dotted lines), standard deviations (horizontal axis; blue dotted arcs), and centred root mean squared errors (solid grey arcs) for summer mean convective parameters from historical HighResMIP simulations (symbols) with ERA5 parameters as the observational reference (open circle with correlation of 1, standard deviation of 1, and a centred root mean squared error of 0). Convective parameters include BS_06km, MU_CAPE, MU_CIN, MU_LCL_HGT, SRH_1km, SRH_3km, and RH_05km. The observational reference for each combination of HighResMIP model and parameter is the corresponding ERA5 convective parameter, with values scaled to have unit standard deviation; climate model parameters are expressed in terms of ERA5 standard deviation units.

To check the technical validity of the HighResMIP convective parameters, a subset of relevant indices^12^, including bulk wind shear from 0-6 km (BS_06km), MU_CAPE, MU_CIN, height of the MU_LCL (MU_LCL_HGT), storm relative helicity at 1 km (SRH_1km), storm relative helicity at 3 km (SRH_3km), and RH_05km, from HighResMIP simulations are compared with those calculated from ERA5. This is done for the summer season when convective storms are most active and is focused on North American land areas for the historical period (2001-2020; +1°C GWL). As shown in Fig. 6, the HighResMIP models capture the broad spatial patterns of these convective parameters relative to ERA5, but they differ in terms of spatial correlation, spatial variability, and overall error. EC-Earth3P-HR (all realizations) ranks highest, consistently showing performance near ERA5 for most convective parameters. Notably, centred root mean squared errors are less than 0.5 standard deviations for all parameters except MU_CIN. The two MRI-AGCM3-2 simulations also perform reasonably well across most parameters, with the 20-km version (MRI-AGCM3-2-S) outperforming the 60-km version (MRI-AGCM3-2-H) in all cases. CMCC-CM2-VHR4 often exhibits larger discrepancies in spatial correlation and standard deviation – particularly noticeable for SRH_1km and MU_CIN, respectively – leading to lower agreement with ERA5 relative to the other models. In general, helicity fields show a greater relative error and a lower spatial correlation than other convective parameters, probably due to coarser vertical sampling in some HighResMIP simulations (8 archived levels for some models; Table 1). (As noted in the technical validation of the ERA5 convective parameters, reduced vertical sampling also leads to infrequent but relatively large deviations in MU_CIN.) Users should interpret helicity diagnostics with appropriate caution and, where feasible, consider complementary kinematic measures (e.g., bulk shear), which should also be inspected for robustness and fitness for purpose.

**Fig. 7:**
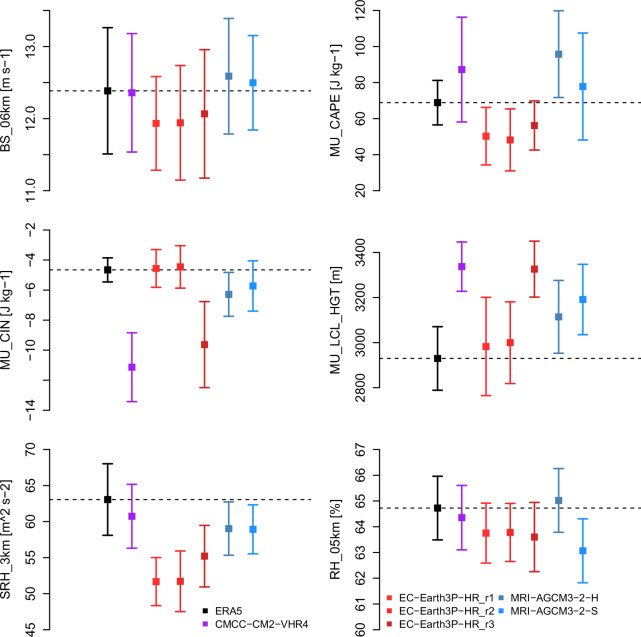
Bias in HighResMIP summer mean convective parameters over North American land areas with respect to ERA5 (+1^°^C GWL; 2001-2020). The central value is the climatological mean; error bars indicate interannual variability (+/ −2 standard deviations of the annual mean values).

Results summarized in Fig. 6 (spatial correlation, standard deviation, and centred root mean squared error) are insensitive to systematic errors with respect to ERA5. Past work has shown that climate model biases can strongly influence proxies of the thunderstorm environment. For example, surface moisture and temperature biases in CMIP6 models over North America are associated with substantial positive biases in CAPE^50^. Figure 7 compares climatological mean values of convective parameters from HighResMIP for the 2001-2020 (+1° GWL) period with those from ERA5; for reference, spread due to interannual variability ( + / −2 standard deviations of the annual mean values) is also shown. A coherent moisture/CAPE bias is not apparent in the HighResMIP models. Instead, biases tend to be model-dependent rather than uniform in sign. Some HighResMIP simulations lie close to ERA5 across multiple parameters, while others exhibit larger offsets. A notable pattern is that simulations with fewer archived pressure levels (Table 1) tend to show larger mean biases, consistent with a greater sensitivity of the parcel-based and layer-integrated diagnostics to vertical sampling.

**Fig. 8:**
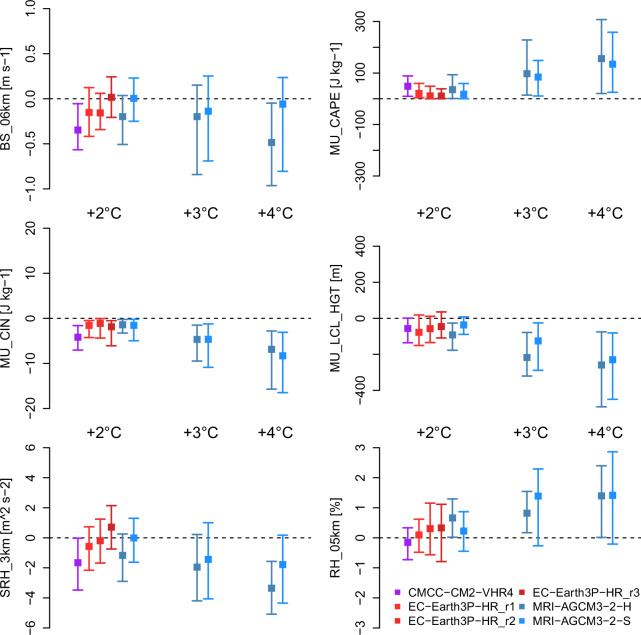
Projected changes (relative to  +1°C GWL; 2001-2020) in summer mean convective parameters over North American land areas as a function of GWL. The central value is the median change over all grid cells, with error bars extending from the 25th to 75th percentiles of the grid cell values.

Although it is not possible to directly validate the HighResMIP projections, future trends on large scales can be evaluated for consistency with projections from coarser-resolution CMIP6 models^12^. Future projections of summer convective parameters over the North American domain (Fig. 8) consistently show increases in instability (MU_CAPE) across all HighResMIP models under +2°, +3°, and +4°C GWLs, accompanied by a stronger cap inhibiting convection (MU_CIN). Robust increases in humidity (RH_05km) and lowering of LCL height (MU_LCL_HGT) emerge under higher GWLs for the MRI-AGCM3-2 model variants. In parallel, deep wind shear (BS_06km) and storm-relative helicity (SRH_3km) decrease as warming intensifies, but there is considerable spread in the near-term among the simulations. These projected changes are generally consistent with those reported for coarser-resolution CMIP6 models^12^, which similarly show robust increases in thermodynamic parameters (CAPE and CIN), while kinematic parameters (deep shear and helicity) exhibit greater spatial and inter-model spread. Overall, the higher resolution in HighResMIP does not fundamentally alter these core signals, but potentially refines the spatial detail.

Given model-dependent historical biases but relatively robust changes projected for the future, there is the potential to apply bias correction to the convective parameters. For example, when training statistical or machine learning models, as in the next section, convective parameters from ERA5 that are used as predictors would typically each be standardized (e.g., to zero mean and unit standard deviation). In this case, when climate model predictors are used to make projections, they would be standardized based on their own model-dependent historical climatological statistics–effectively a form of bias correction. In general, care must be taken because bias adjustment of derived nonlinear, multivariable indices (e.g., CAPE, CIN, etc.) can affect the climate change signal, with the effect depending on how the correction is applied^51^. For example, the projected change in an index calculated from biased state variables may be different than that found after correcting the input state variables first and then recomputing the index.

### Links between parameters and lightning

To make accurate and robust predictions using statistical or machine learning models, they should be trained on informative input-output pairs. In the context of modelling thunderstorms using CanCPLD, this involves identifying convective parameters that are relevant for modelling the occurrence or intensity of lightning at a given time and location, both historically and under future climate change conditions. In other words, there should be physically-informed, stable relationship between each potential predictor and lightning^12^. As an example, ECCC’s Canadian Atmospheric Model (CanAM)^52^, the atmospheric component of the Canadian Earth System Model (CanESM)^53^, now incorporates a lightning parameterization scheme based on the logistic regression equation (EB21-LR)^54^

1 $$\begin{array}{c}\,{\rm{logit}}(p(s))={\beta }_{0}+{\beta }_{1}{\rm{CAPE}}(s)+{\beta }_{2}{\rm{LCL}}(s)+{\beta }_{3}r(s)+{\beta }_{4}{\rm{CAPE}}(s)\times {\rm{LCL}}\,(s)\\ \,\,\,\,\,\,+\,{\beta }_{5}\,{\rm{CAPE}}(s)\times r(s)+{\beta }_{6}{\rm{LCL}}\,(s)\times r(s)\end{array}$$

where logit(⋅) is the inverse of the standard logistic function, *p*(*s*) is the probability of lightning occurrence at space-time location *s*, CAPE is the standardized (to zero mean and unit standard deviation) convective available potential energy, LCL is the standardized lifting condensation level, *r* is the standardized column saturation fraction, and *β*_0…6_ are logistic regression coefficients^55^. This scheme has demonstrated good performance over global land and ocean^54,55^, and is based on a subset of standard convective parameters that are included in (or can easily be calculated from) CanCPLD.

**Fig. 9:**
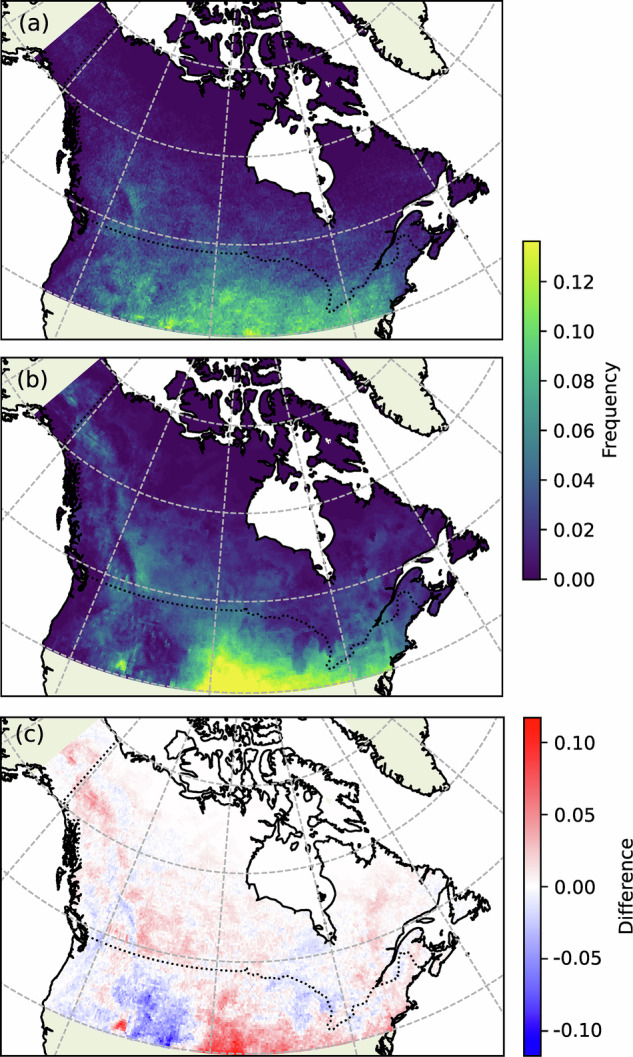
(**a**) Frequency of days with lightning, expressed as a proportion of the year, detected by the CLDN at each grid cell in 2004. (**b**) Frequency of days with lightning predicted for 2004 using the logistic regression model formulation by Etten-Bohm *et al*. (2021) (EB21-LR)^54^. (**c**) EB21-LR predicted frequency minus CLDN observed frequency.

As a check on the technical validity of the link between the ERA5 convective parameters and the lightning data in CanCPLD, a logistic regression model based on equation (1) is trained for high latitude regions of North America. The goal is not to construct an optimal model but rather to demonstrate that a reasonable statistical model can be obtained using CanCPLD. For training, daily mean ERA5 convective parameters from 2003 are used as predictors, and concurrent CLDN lightning occurrence data (binary flag), aggregated to the same grid as ERA5, are used as the targets. CAPE and LCL are represented by MU_CAPE, MU_LCL_HGT. As *r* is not explicitly calculated by sounding_compute, it is approximated here by taking the mass-weighted mean of RH_01km, RH_14km, and RH_36km. Predictor variables are standardized using mean and standard deviation values from the 2003 training data. The probability threshold for positive lightning prediction is determined by optimizing the F1 score – the harmonic mean of precision and recall – of the EB21-LR model, with data from 2002 serving as the validation set. Predictions of lightning occurrence are then made for a 2004 test set. Figure 9 compares the annual frequency of days with lightning in 2004 predicted by the trained model with those observed by the CLDN. Although there are areas of overprediction and underprediction, spatial correlation is high (0.86) and error magnitudes – noting differences in training data and domain – over common areas of the US are comparable to those of CanESM (cf. Fig. 1)^55^.

## Complementary Datasets

For users requiring very high-resolution data, convection-permitting simulations can serve as useful complements to the ERA5/HighResMIP framework of CanCPLD. From an observational perspective, the NCAR-USGS CONUS404 reanalysis^56^ (4-km, hourly) provides a long, internally consistent dataset based on dynamical downscaling of ERA5 using the WRF model. Strengths include explicit representation of deep convection at convection-permitting scales, realistic orographic effects, and hourly fields suitable for process studies. However, the spatial coverage is focused on the conterminous United States, with only partial extension into southern Canada. Furthermore, the record ends in September 2021. These features make CONUS404 well suited for targeted case studies and process diagnostics^7^ where there is spatial/temporal overlap, but without the ability to investigate high latitude regions.

For climate projections, the companion NCAR CONUS II dataset^57^ (4-km, hourly) offers recent-past (1996-2015) |and late-century (2080-2099) transient climate simulations by WRF for a domain that extends across much of North America, including most of Canada. These simulations are based on a single realization of internal climate variability and do not provide an observation-synchronous chronology within the historical period. Consequently, CONUS II is best viewed as a complementary resource for evaluation, process studies, and sensitivity tests, rather than as the primary basis for historical climatological analyses or comprehensive future projections.

Taken together, these WRF products provide a convection-scale perspective where coverage and periods align, while the ERA5 and HighResMIP components of CanCPLD supply continental-scale consistency, an observation-anchored historical context, and a multimodel framework for future projections.

## Usage Notes

The CanCPLD dataset is provided in self-describing netCDF files, which contain metadata describing variables, units, and dimensions, ensuring compatibility with widely used scientific analysis software such as Python, R, and MATLAB, and ease of conversion to cloud-ready binary storage formats like Zarr^58^. Given the high spatiotemporal resolution, users should anticipate significant data volumes. Computational tools capable of efficiently processing such large datasets are recommended.

Temporal inhomogeneities may exist in the historical lightning data due to improvements in detection technology, especially for intra-cloud flashes, and in the ERA5 convective parameters due to changes in assimilated observations. Users should be aware of the limitations of reanalyses in representing convective parameters^27,48,49^, especially compared to those derived from rawinsonde observations.

Climate model projections from HighResMIP are adopted for their relatively high spatial resolutions, which closely match ERA5, ensuring some degree of consistency when comparing historical observations and simulations. However, this focus on high resolution comes with trade-offs. The ensemble size is relatively small, and some HighResMIP models only run through mid-century, limiting the available data for higher GWLs. As a result, users should be aware of these constraints when interpreting projections, especially limitations in the ability to characterize structural uncertainty. Furthermore, differences in horizontal and vertical resolution – the number of archived pressure levels (Table 1) – between ERA5 and HighResMIP outputs may also affect comparative analyses and require careful consideration.

Finally, the lightning and ERA5 datasets in CanCPLD are expected to be extended in time annually to increase the sample size available to train statistical and machine learning models. As new high-resolution climate model simulations become available, additional projections may also be added to increase the ensemble size and allow projection uncertainty to be better characterized.

## Supplementary information

Supplementary Table 1

## Data Availability

Convective parameters, lightning data, and grid cell areas that form CanCPLD^47^ are available from the ECCC Data Catalogue with identifier 10.18164/9a68a501-9a87-441a-8073-980ae68438ef and also from the Government of Canada Open Data Portal (https://open.canada.ca/data/en/dataset/9a68a501-9a87-441a-8073-980ae68438ef).

## Code availability

This study used the the sounding_compute routine from version 1.1.3 of the thundeR R package^45,46^ to calculate convective parameters; thundeR is distributed under the GPL-3 license and is available for download from https://cran.r-project.org/package=thunder. This version of the R package and code used to develop CanCPLD are available in the Zenodo repository with the identifier 10.5281/zenodo.14681048^59^.
